# A Sizeable Adrenal Ganglioneuroma: A Case Report

**DOI:** 10.7759/cureus.44611

**Published:** 2023-09-03

**Authors:** Ehab M El Hosseny Sadek, Salem Bashawieh, Mohammed Almasabi, Abrar Najjar, Banan Najmi, Ahmed S AL Zomia

**Affiliations:** 1 Surgical Oncology, King Abdullah Medical City, Makkah, SAU; 2 General Surgery, King Abdulaziz Hospital, Makkah, SAU; 3 Medicine, King Khalid University, Abha, SAU

**Keywords:** adrenal mass, urinary metanephrine, mibg, hypertension, ganglioneuroma

## Abstract

Adrenal ganglioneuromas are mostly asymptomatic, although they may manifest with compressive local effects. We present a 27-year-old man with no medical history who was referred to the surgical oncology clinic due to the incidental finding of a left adrenal mass. The initial computed tomography (CT) abdomen revealed a large mass causing displacement of adjacent organs. A CT-guided biopsy was inconclusive, and further evaluation with an NM endo-adrenal (MIBG) medullary scan pointed to a possible diagnosis of pheochromocytoma. Laboratory tests showed normal levels of urinary metanephrine and normetanephrine. The patient's history revealed chronic abdominal pain, with no symptoms of hypertension, headache, palpitations, or sweating. Subsequently, the patient underwent a left adrenalectomy without complications. This case underscores the importance of a comprehensive approach in managing adrenal masses, particularly when dealing with non-specific symptoms, emphasizing the importance of timely diagnosis and appropriate treatment.

## Introduction

Adrenal ganglioneuroma (AGN), a benign tumor originating from the autonomous sympathetic nervous system, can be found at various sites in proximity to autonomic ganglia, including the adrenals, retroperitoneum, mediastinum/thorax, and cervical area [1, 2]. It is considered the least aggressive tumor among all neuroplastic tumors due to its exclusive presence of mature cells, making it a rare entity within this category [3]. Typically, these tumors are discovered in patients over the age of 10 years and are often asymptomatic, although they can manifest with local compressive effects [4] and occasionally with symptoms similar to pheochromocytoma such as palpitation, sweating, and hypertension [5]. The diagnosis of GN is challenging; the misdiagnosis rates for AGN based on CT and magnetic resonant imaging (MRI) scans are 64.7%. In addition, clinicians and surgeons frequently lack sufficient knowledge about this rare condition [6]. Adrenalectomy is considered the gold-standard treatment for these tumors [4]. The estimated incidence of ganglioneuromas (GNs) is around one occurrence per million individuals [7]. The most extensive analysis to date on GNs, irrespective of age, was issued in 2021. This study included cases published on PubMed between 1995 and 2018, revealing a total of 364 cases. Among these cases, 65.7% pertained to adults and there was a prevalence of women (62%). Incidental discovery occurred in 24.5% of all cases, the most common locations being the abdomen/pelvis (66.2%) followed by GNs in the adrenal region (32.1%). The occurrence of postoperative complications was notably low after adrenalectomy, at just 1%, especially when compared to cases situated in the head, neck, and thorax. Furthermore, no cases of recurrence were reported, which confirms the consistently benign nature of GN regardless of age [8].

## Case presentation

History and clinical examination

**Figure 1:**
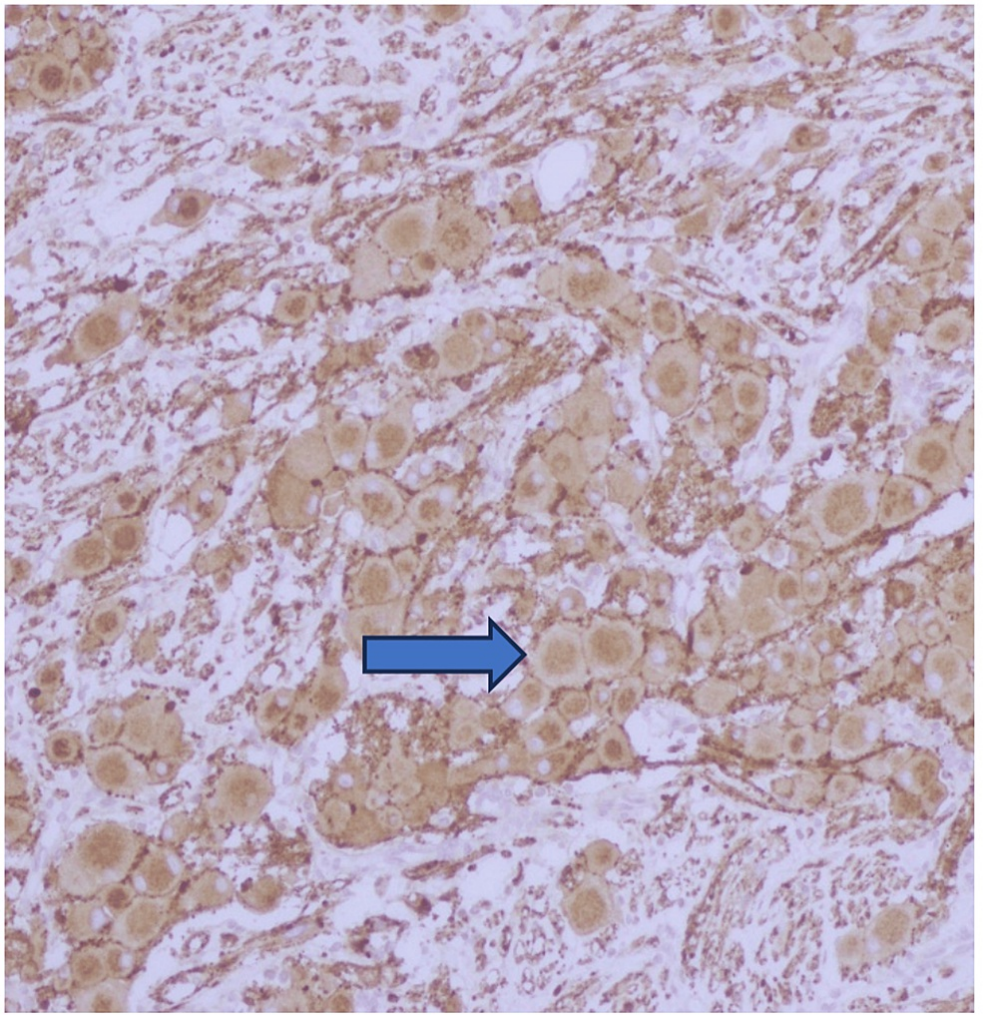
Both Schwann cells/stroma and ganglion cells are positive with synaptophysin immunostain.

**Figure 2:**
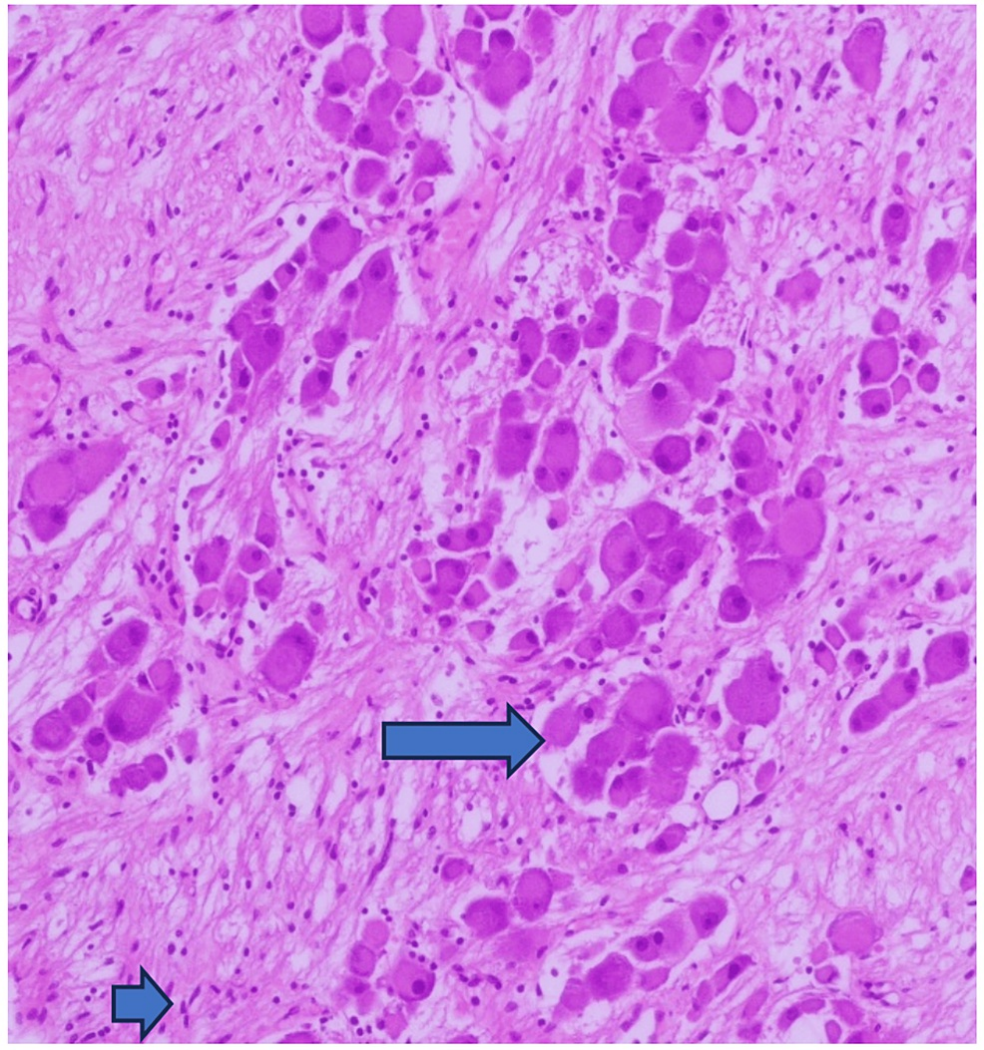
Many mature ganglion cells seen having compact, eosinophilic cytoplasm with distinct cell borders, single eccentric nucleus and prominent nucleolus. In the background these are mixed with Schwann cells arranged in small intersecting fascicles.

A 27-year-old male with no known medical history was referred to the surgical oncology clinic for a left adrenal mass. He weighed 76 kg and measured 176 cm (body mass index = 21.63). The general examination, including assessments of blood pressure, respiratory rate, pulse, and temperature, yielded normal results. Furthermore, the blood parameters and the blood composition exhibited normal values. A computed tomography (CT) abdomen performed in a primary hospital revealed the presence of a left adrenal mass. A CT-guided biopsy was subsequently performed, and the results showed a spindle cell neoplasm versus gastrointestinal stromal tumor (GIST) (Figures 1, 2).

During the examination in the surgical clinic, the patient only complained of chronic abdominal pain, denying any history of hypertension, headache, palpitations, or sweating. Laboratory tests showed urinary metanephrine (1.12 Umol/day. Mcg/24h) and normetanephrine (1.3 Umol/day. Mcg/24h) within normal limits. The abdominal CT abdomen on 9/4/2018 revealed a large mass in the left upper abdominal quadrant, causing an anterior and superior displacement of the spleen, an anterior displacement of the pancreas and stomach, and an inferior displacement of the left kidney. The mass measures approximately 15.9 x 11.8 x 13.5 cm. The left adrenal gland was not visible, and the mass appeared to be separate from the spleen, pancreas, and left kidney. Internal vascular structures were evident in the arterial phase, with an average density of 44 HU in the Porto venous phase. An incidental note revealed a double inferior vena cava (IVC) with the left renal vein draining into the left IVC. There was a suspicion of a small thrombus within the left renal vein (4/56).

**Figure 3:**
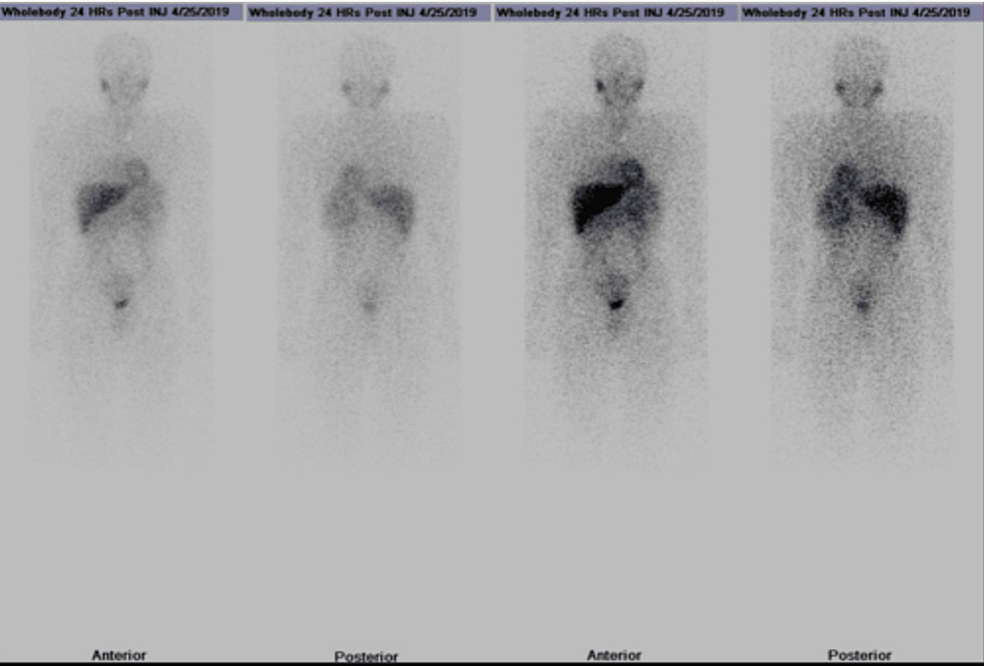
The MIBG scan revealed a focal area of increased tracer uptake in the left upper abdomen, corresponding to a large hypodense lesion in the left upper quadrant with elevated tracer activity.

On April 25, 2018, an NM endo-adrenal (MIBG) medullary scan was performed, involving the injection of approximately 60 MBq of 131I-MIBG and iodine overload using Lugol 5%® solution for seven days. Whole body planar images and spot views of the chest were obtained 24 hours after injection, along with SPECT/CT of the abdomen. The scan revealed a focal area of increased tracer uptake in the left upper abdomen, corresponding to a large hypodense lesion in the left upper quadrant with elevated tracer activity. Physiological activity was observed in various regions and no other abnormal focal tracer activity was observed. The findings were consistent with an MIBG-avid lesion, indicative of an adrenergic neuroectodermal tumor, likely pheochromocytoma (Figure 3). The MIBG scan revealed a focal area of increased tracer uptake in the left upper abdomen, corresponding to a large hypodense lesion in the left upper quadrant with elevated tracer activity.

Treatment

The patient was referred to the endocrinology department for further evaluation of any endocrinopathy. The impression from the endocrinology evaluation suggested a likelihood of a non-secretory neuroendocrine tumor. The proposed plan included ensuring that there were no significant endocrine issues related to the surgery. Although the risk of hypertensive conditions during the operation was considered very low, the anesthesia team was advised to be prepared to manage such situations. Additionally, the presence of a renal vein thrombus required close monitoring and management by the primary medical team and the vascular team.

**Figure 4:**
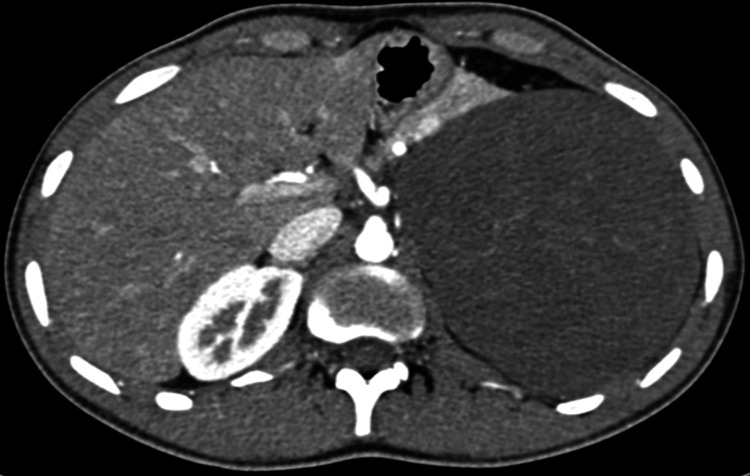
CT before surgical resection of the tumor. Large mass present in the left upper abdominal quadrant, causing anterior and superior displacement of the spleen, anterior displacement of the pancreas and stomach, and inferior displacement of the left kidney. It measures approximately 15.9 x 11.8 x 13.5 cm. The left adrenal gland is not visualized. The mass appears to be separable from the spleen, pancreas and left kidney. The mass shows internal vascular structures in the arterial phase with an average density of 44 HU in the porto-venous phase.

The patient was recommended to undergo surgical resection. On July 1, 2019, a left adrenalectomy was performed, which was uneventful. Postoperatively, the patient was admitted to the intensive care unit (ICU) and later moved to the ward. Following a smooth recovery, the patient was discharged in good condition, with no complaints, and scheduled for regular follow-up appointments. The histopathology report revealed a diagnosis of GN (Figure 4).

Prognosis

**Figure 5:**
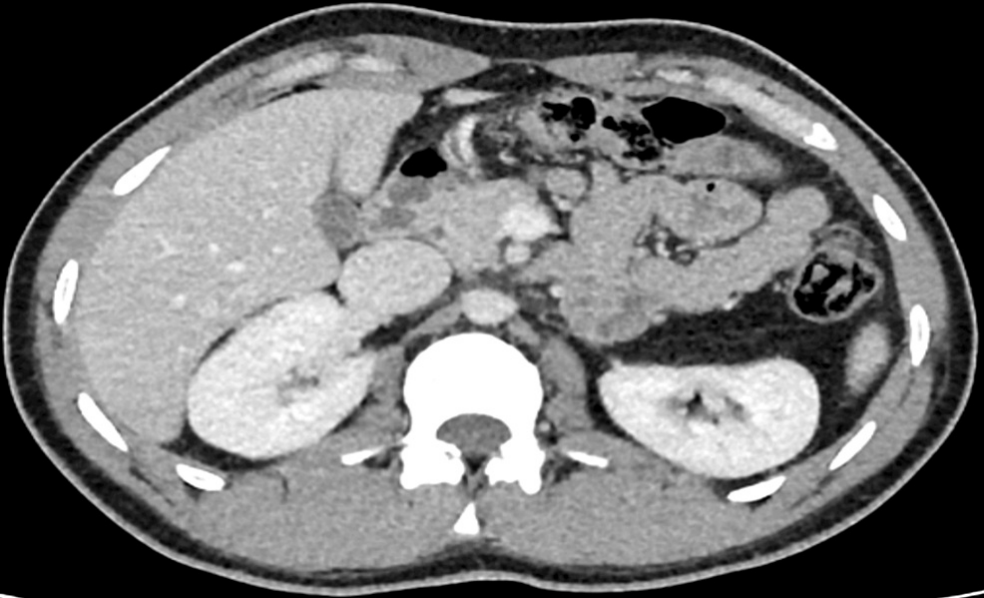
CT after surgical resection of the tumor. Kidneys are of normal enhancement with no stone, mass or hydronephrosis. Right adrenal is normal, while the left adrenal is resected with no obvious residual masses in the surgical bed.

Subsequent CT scans of the abdomen and pelvis conducted on February 12, 2023 showed no evidence of local recurrence or distant metastasis, providing a positive outlook for the patient's health (Figure 5).

## Discussion

Adrenal gangliogliomas generally exhibit a slow growth pattern and often remain asymptomatic. However, our case stands out as it involves a massive AGN in a male patient who presented nonspecific flank pain. Interestingly, the initial CT did not diagnose the adrenal mass. This highlights the importance of a thorough evaluation in patients with persistent flank pain. Our case presented a relatively large tumor, measuring 15.9 cm in diameter. It should be noted that the largest reported ganglioneuroma to date measured 19 cm in size [9].

The differential diagnosis for the observed adrenal mass includes several possibilities: pheochromocytoma, adrenocortical carcinoma, and metastatic adrenal mass are potential considerations. In addition, other potential diagnoses include adrenal myelolipoma, ganglioneuroma, and neuroblastoma. AGN and neuroblastoma are also part of this category. The distinguishing ganglioneuroma from these two tumor types can be achieved by the presence of mature sympathetic ganglion cells. Generally, AGNs are nonfunctioning tumors, but in some cases, they may be associated with pheochromocytoma and secrete catecholamines and their metabolites. There have been rare suggestions in the literature that AGNs can also secrete cortisol and androgen [10]. In our particular case, the patient exhibited normal catecholamine levels, indicating the presence of a non-functioning GN. The primary symptom upon presentation was the presence of an abdominal mass.

It has been suggested that adrenal masses smaller than 3 cm have a very low probability of being functional, while those larger than 3 cm are more likely to be functional. Therefore, the endocrine evaluation should be considered for large adrenal masses. Additionally, when an adrenal mass is discovered, 75% of cases are due to metastases from other organs [11]. The primary sites of metastasis are typically the kidney, colon, breast, esophagus, pancreas, liver, and stomach. Adrenal masses caused by metastases are usually bilateral [11].

The MRI and CT views of AGNs can resemble those of other adrenal tumors. Consequently, based solely on imaging techniques, distinguishing ganglioneuroma from adrenocortical carcinoma and pheochromocytoma is challenging. In cases of adrenal incidentalomas, the urine should be monitored for catecholamines and fractionated metanephrine for 24 hours prior to surgery. Elevated levels of metanephrine and/or catecholamine indicate the presence of pheochromocytoma with a sensitivity and specificity of 91% and 98%, respectively [12]. The fine needle aspiration biopsy is not very reliable in ruling out malignancy, as it has a high rate of false negative results and can present complications. In the case of our patient with a relatively large tumor, open surgery was the preferred primary treatment modality for adrenocortical carcinoma.

## Conclusions

The differentiation of AGNs from other adrenal tumors through imaging techniques, such as MRI and CT, can be challenging. AGNs are distinct from neuroblastomas and are characterized by the presence of mature sympathetic ganglion cells. When dealing with adrenal masses, an endocrine evaluation is essential for masses larger than 3 x 3 x 3 cm, which are more likely to be functional.
